# Crystal structure and metallization mechanism of the π-radical metal **TED**†

**DOI:** 10.1039/d0sc03521a

**Published:** 2020-09-11

**Authors:** Yuka Kobayashi, Kazuto Hirata, Samantha N. Hood, Hui Yang, Aron Walsh, Yoshitaka Matsushita, Kunie Ishioka

**Affiliations:** National Institute for Materials Science (NIMS) Sengen 1-2-1 Tsukuba Ibaraki Japan kobayashi.yuka@nims.go.jp; Department of Materials, Imperial College London Exhibition Road London SW7 2AZ UK a.walsh@imperial.ac.uk

## Abstract

Radical electrons tend to localize on individual molecules, resulting in an insulating (Mott–Hubbard) bandgap in the solid state. Herein, we report the crystal structure and intrinsic electronic properties of the first single crystal of a π-radical metal, tetrathiafulvalene-extended dicarboxylate (**TED**). The electrical conductivity is up to 30 000 S cm^−1^ at 2 K and 2300 S cm^−1^ at room temperature. Temperature dependence of resistivity obeys a *T*^3^ power-law above *T* > 100 K, indicating a new type of metal. X-ray crystallographic analysis clarifies the planar **TED** molecule, with a symmetric intramolecular hydrogen bond, is stacked along longitudinal (the *a*-axis) and transverse (the *b*-axis) directions. The π-orbitals are distributed to avoid strong local interactions. First-principles electronic calculations reveal the origin of the metallization giving rise to a wide bandwidth exceeding 1 eV near the Fermi level. **TED** demonstrates the effect of two-dimensional stacking of π-orbitals on electron delocalization, where a high carrier mobility of 31.6 cm^2^ V^−1^ s^−1^ (113 K) is achieved.

## Abstract 2

The molecular arrangement that enables metallic conduction in a single-component pure organic crystal is revealed by single-crystal X-ray diffraction.

## Graphical abstract

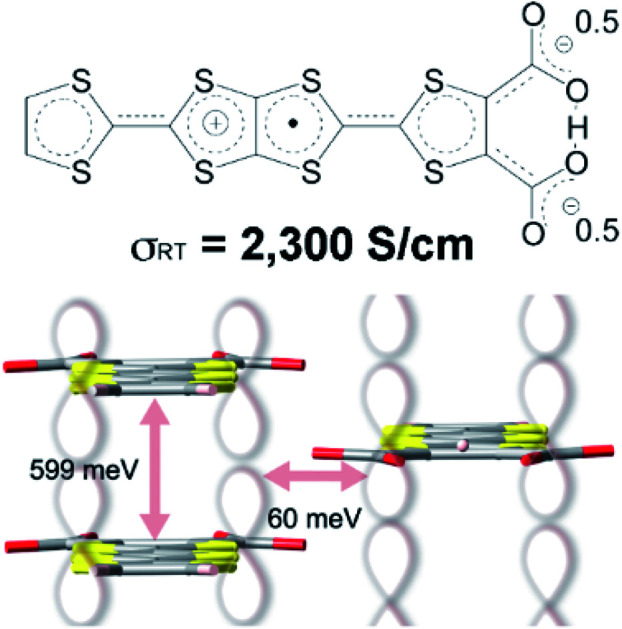

Organic molecular solids are typically insulating due to their paired electrons in spatially localized s- and p-orbitals. The concept of charge-transfer (CT) between donor and acceptor^1^ enabled the development of conducting molecular complexes (salts) including semiconducting perylene-bromine,^2^ metallic tetrathiafulvalene (TTF)-tetracyano-*p*-quinodimethane (TCNQ),^3^ and polyacethylene doped with halogen molecules.^4^ A different strategy was proposed in the 1970s based on organic radicals with an open-shell electronic structure.^5^ π-Radicals such as neutral-,^6^ fully-conjugated^7^ and zwitterionic (betainic)^8^ molecules, with an unpaired electron in their singly occupied molecular orbital (SOMO), offered potential candidates. However, all these π-radicals were insulators or semiconductors with a finite bandgap, which is due to the SOMO being localized on an individual molecule. In the Mott–Hubbard model,^9^ the case of the π-radical solids can be described by the on-site Coulomb repulsion *U* being larger than the electronic bandwidth *W* (*U*/*W* > 1), in contrast to *U*/*W* < 1 in molecular metals like CT metal systems (Fig. 1a).

**Fig. 1:**
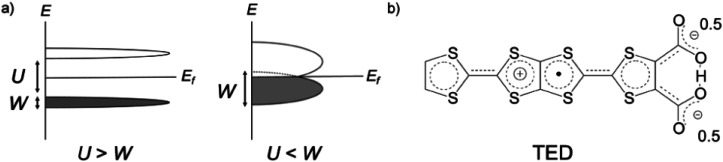
(a) Schematic representation of the electronic band structure of Mott–Hubbard insulators (left) and possible molecular metals (right), respectively. Solids formed from typical π-radicals possess large on-site Coulomb repulsion *U* compared with the electronic bandwidth *W*, resulting a finite bandgap (left). This requires a new mechanism to expand *W* and overcome *U* to achieve a metallic state at ambient pressure in π-radical crystals. (b) Molecular structure of the zwitterionic radical, tetrathiafulvalene-extended dicarboxylate (**TED**) with a symmetric intramolecular hydrogen bond.

A straightforward approach to realize high conductivity in π-radical systems is to enhance the intermolecular interaction by applying high pressure.^10^ Bisdithiazolyl radical crystal achieved *W* ∼ 1 eV near the Fermi level and the room temperature conductivity *σ*_RT_ = 2 S cm^−1^ under 11 GPa pressure.^10*c*^ An alternative route is to decrease the interatomic spacing by incorporating a metal ion. Introduction of a semimetal Se and intermolecular hydrogen bonding in a donor-type radical succeeded to improve a conductivity to *σ*_RT_ = 19 S cm^−1^ but still required high pressure over 1 GPa for breaking its insulating character.^10*d*^ An organometallic compound with a transition metal, [Ni(tmdt)_2_], by contrast, is known to form a three-dimensional (3D) Fermi surface with *W* = 0.48 eV and metallic conduction with *σ*_RT_ = 400 S cm^−1^ at ambient pressure.^11^ A breakthrough concept for expanding *W* at ambient pressure is desired for achieving metallization in pure organic π-radicals.

Tetrathiafulvalene-extended dicarboxylate (**TED**) is an organic air-stable zwitterionic radical (Fig. 1b),^12^ which was designed based on carrier generation induced by a stably-introduced protonic defect (–H^+^) in hydrogen-bonding molecules without adopting CT between multiple molecules.^13^ A polycrystalline film of **TED** exhibited metallic conduction at ambient pressure, but the mechanism has not been clarified yet due to lack of single crystal information.^12^ Herein, we report the first crystal structure and intrinsic electronic properties of the recently grown single crystal **TED**. Structural analysis and quantum chemical simulations based on the single crystal reveal the origin of its metallic behavior.

## Results and discussion

### Crystal structure

**Fig. 2:**
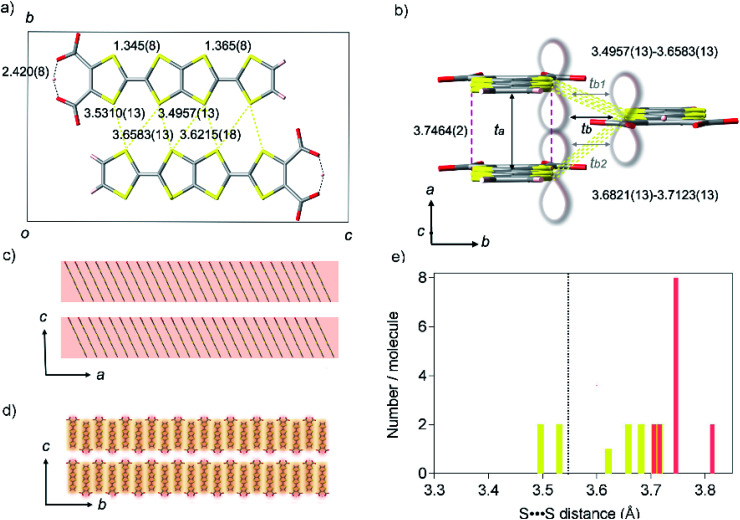
Crystal structure of **TED**. (a) A unit cell projected on the *a*-axis, represented with black square, consisting of two **TED** molecules with intermolecular distances of S⋯S contacts (yellow dotted line) and intramolecular hydrogen bond (black dotted line) in unit of Ångstrom. (b) A molecular stacking unit along the *a* and *b*-axes. Red break and yellow dotted lines show face-to-face and side-by-side intermolecular distances of S⋯S contacts, where π-orbital orbitals overlap longitudinally along the *a*-axis and transversely along the *b*-axis, respectively, as is guided with the lobe image. Black arrows display the transfer integrals *t*_*a*_ (599 meV), *t*_*b*_ (60 meV), which is averaged [*t*_*b*_ = (*t*_*b*1_ (69 meV)+ *t*_*b*2_ (51 meV))/2]. (c and d) Super-structures of the **TED** molecular units crossing the *c*-axis; (c) stacking along the *a* direction; electric conduction occurs predominantly within the **TED** molecular layer, (d) stacking along the *b* direction. Red and yellow shadows show the anisotropic conduction pathways with *t*_*a*_ and *t*_*b*_, respectively, in contrast, inter-layer conductions along the *c*-axis are much lower with *t*_*c*_ ∼ 0. (e) Distribution of S⋯S distance in one molecule with an indication of van der Waals sum distance (black dotted line). Only one site was counted in the nearest neighbors with the same symmetry operation. Red and yellow bars show the contact along the *a*-axis and the *b*-axis, respectively.

**TED** was synthesized by a protonic-defect induced carrier generation *via* a solution process,^13^ as well as previously reported self-standing film.^12^ Single crystals were slowly grown over several months from organic solvent without an electrocrystallization technique to have a typical dimension of 400 × 20 × 10 μm^3^ (see Experimental methods). The micron-order single crystals and nano-size polycrystals obtained in the self-standing film^12^ are isomorphous (ESI, Fig. S1 and S2†). The crystal structure of **TED** was solved from the single-crystal X-ray diffraction (SXRD) data at 113 K. The structure belongs to a space group *P*2_1_/*m* (ESI, Tables S1, S2 and Fig. S3†).^14^ The unit cell, shown in Fig. 2a and b, consists of two planar **TED** molecules lying side by side with their long axis along the *c* direction and with an offset between them in the *a* direction. The intermolecular distance within the unit cell is 3.4957(13) Å. The unit cells stack along the *a-*axis with an interplanar distance of 3.7464(2) Å and along the *b-*axis with 3.6821(13) Å, as shown in Fig. 2b, to form a stacking **TED** molecular layer. The stacked **TED** layers align themselves crossing the *c*-axis with a small vacant space between them, as shown in Fig. 2c and d. It should be noted here that the small vacancy between the **TED** layers sometimes intercalates solvent (DMSO and/or H_2_O). The solvent does not have remarkable intermolecular interaction such as hydrogen bonds with **TED**, but it affects conductivity. In this study, only the solvent-free crystal (**TED** crystal without solvent) is reported in order to focus on a pure **TED** system excluding the effect of solvent intercalation.

Two central C=C bond distances in **TED** are close to each other; 1.345(8) and 1.365(8) Å (ESI, Fig. S4†). The values are similar to those in traditional CT metals, 1.35(2) and 1.36(2) Å for (BDT-TTP)_2_SbF_6_ salt.^15^ C–S bond distances are also quite close to each other in the two TTF moieties, different from those of calculated monomer structure with a localized radical electron on one side of TTF with carboxyl groups.^12^ Moreover, a symmetric intra-molecular hydrogen bond is formed with O⋯O distance of 2.420(8) Å in the carboxylates, which is categorized in short-strong hydrogen bond (SSHB) or low-barrier hydrogen bond (LBHB) possessing a centered proton with a single-well potential.^16*a*^ SSHB has a covalent nature coupled with π-delocalization,^16*b*^ which plays an important role to delocalize and stabilize the radical electron in a **TED** molecule. These facts support the radical electron widely spreads on a whole **TED** molecule through π-conjugation, not only on one side of the TTF moieties, which is advantageous in charge transport. The dominant intermolecular interaction in the **TED** crystal arises from the S⋯S contacts along the *b*-axis (side-by-side contact), whose seven nearest-neighbor distances range from 3.4957(13) to 3.6583(13) Å, close to their van der Waals sum of 3.6 Å.^17^ The distance between facing π-planes along the *a*-axis (face-to-face contact) is even larger, from 3.7059(13) to 3.8127(13) Å. The S⋯S separations in the **TED** crystal are mostly longer than the van der Waals sum (Fig. 2e) and we find that even the minimum distance 3.4957(13) at the side-by-side contact is not very short comparing with 3.350(2) Å of an organometallic metal [Ni(tmdt)_2_].^11*b*^

Quantum chemical analysis on the experimental results revealed strong anisotropy in the wavefunction overlap, *t*_*a*_ = 599 meV and *t*_*b*_ = 60 meV [*t*_*b*_ = (*t*_*b*1_ + *t*_*b*2_)/2], where the *t*_*a*_ and *t*_*b*_ are the longitudinal and transverse transfer integral of the π-orbital (Fig. 2b). The longitudinal transfer integral is comparable to that of highly conductive CT salts.^18^ The stacked **TED** molecular layer is electrically insulating with void interlayer transfer integral along the *c*-axis *t*_*c*_ ∼ 0. These results indicate that the conduction occurs predominantly along the *a*-axis of the **TED** with the interplanar spacing 3.7464(2) Å (Fig. 2c and d), strongly suggesting the effectiveness of π-orbital stacking over short S⋯S contacts in the present case.

### Transport properties

**Fig. 3:**
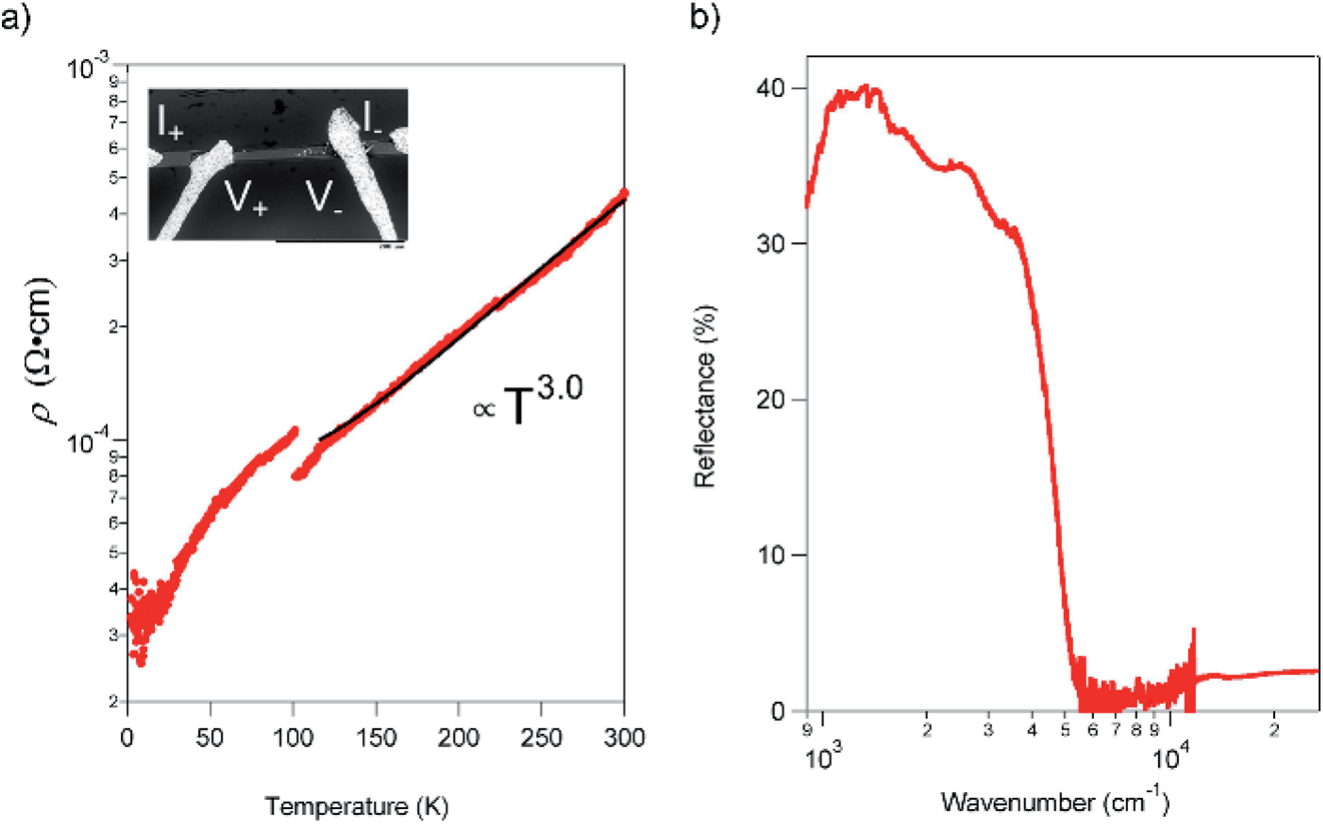
Transport and optical properties of the **TED** single crystal. (a) Temperature dependence of *ρ*, measured with the four-probe method from 300 to 2 K in a He cryostat. The black curve is fitting to a power-law function. Inset shows the scanning electron microscope image of the sample setting with Ag electrodes. The electrodes were attached along the *a*-axis. (b) Reflectance spectrum with P-polarized incident light on the *ab* plane (*E*//*a*) in **TED** single crystal at RT.

The resistivity (*ρ*) within the *ab* plane of the **TED** single crystal samples was examined as a function of temperature with the four-probe method using Ag paste as contact at ambient pressure. The result, shown in Fig. 3a, exhibited metallic conduction down to 2 K. We found no evidence of a Peierls instability in **TED**, which is often observed in low-dimensional π-electron systems.^19^*ρ* is decreased from 435 μΩ cm^−1^ at RT to 33 μΩ cm^−1^ at 2 K; this corresponds to the increase of conductivity *σ* = *ρ*^−1^ from 2300 S cm^−1^ at RT to 30 000 S cm^−1^ at 2 K. In addition, *ρ* exhibited a visible gap at *T* ∼ 100 K and can be fit to *ρ* ∝ *T*^3^ for *T* > 100 K. The great improvement of the conductivity in single crystal compared with the nano-polycrystalline film^12^ and the metallic behavior down to 2 K can be attributed to efficient anisotropic conduction in single crystal with homogenous crystalline axis and reduced scattering at the grain boundaries. The observed super-linear temperature-dependence is in stark contrast to the linear dependence of *ρ* for simple metals.^20^ Moreover, we note that there is a small gap in the *ρ*–*T* plot at around 100 K. It is considered not to be a phase transition to another electronic phase with a drastic change of the electronic state but some modulation of the conduction behavior, which is detectable in high-quality crystals that enable to reflect only intrinsic electronic properties of the **TED** conducting layers in resistivity measurements. We point out that the temperature for the gap (*k*_B_*T* = 8.8 meV) is quite close to the energy of a low-frequency phonon mode at 72 cm^−1^ (*hω* = 8.9 meV), which appears intensely in the Raman spectrum (ESI, Fig. S5†). There may be an unconventional mechanism for the metallic conduction of **TED**, which requires further analyses for clarification.

The reflectance of the crystal exhibited a Drude-type dispersion with a clear plasma edge at around 5000 cm^−1^, as shown in Fig. 3b. We note that the absolute value of reflectance is relatively small (≈40%), possibly due to the surface roughness of the crystal (ESI, Fig. S6†). The free carrier concentration is estimated to be 2.2 × 10^21^ cm^−3^, which is comparable to the CT metal, κ-(BEDT-TTF)_2_I_3_.^21^ The majority carriers are holes due to the cationic charge on the fused rings and as evidence by the positive sign of thermopower.^12^ The mobility (*μ*) of the **TED** single crystal at 113 K, which is the temperature determined the crystal structure, is 31.6 cm^2^ V^−1^ s^−1^ from the conductivity and the carrier concentration. This value is remarkably large compared to typical molecular conductors where *μ* < 1 cm^2^ V^−1^ s^−1^, and comparable to those of the highest class of organic systems.^22^

### Quantum chemical calculations

**Fig. 4:**
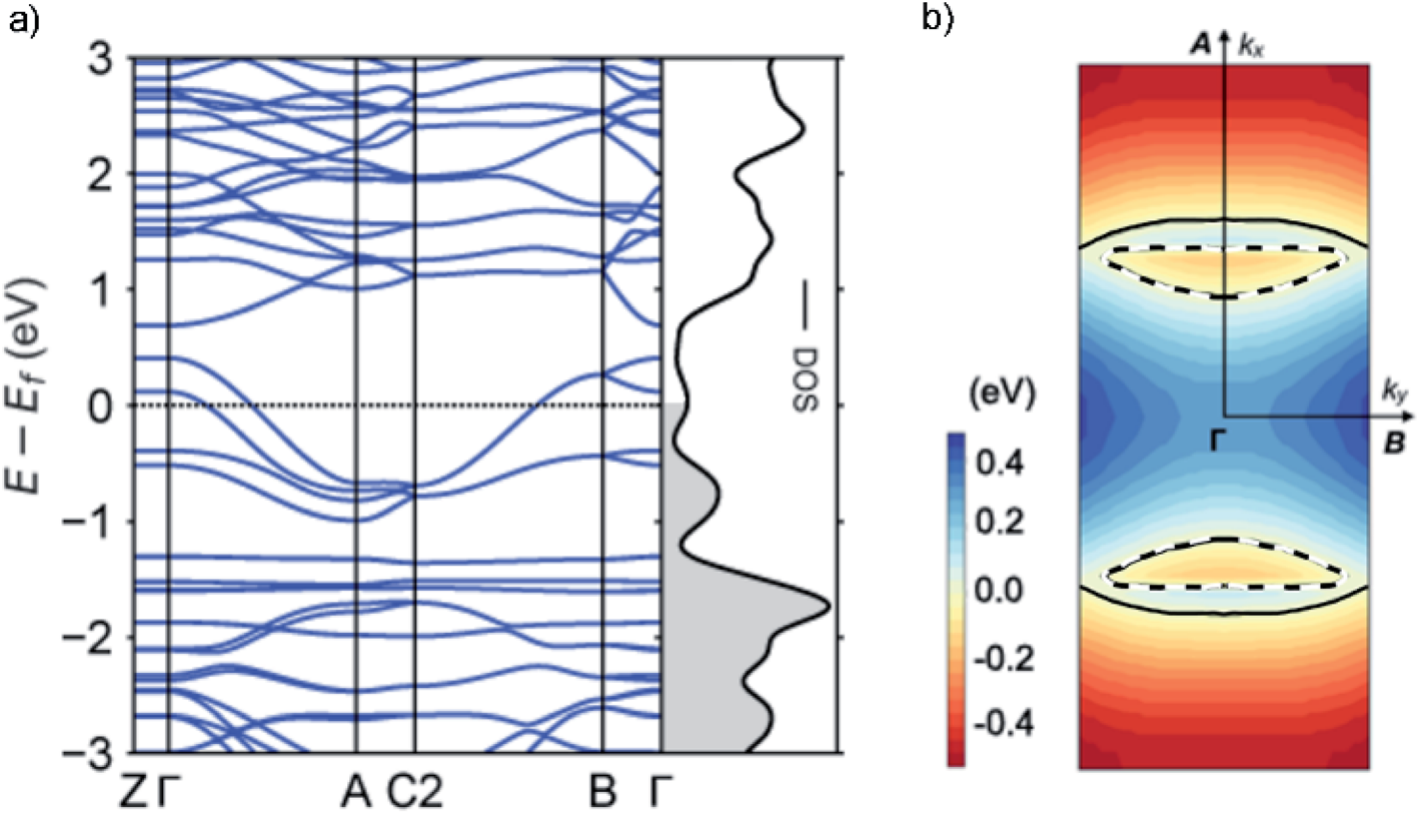
Electronic properties of **TED** calculated by density functional theory based on the single crystal structure. (a) Band dispersion along the high-symmetry lines of the first Brillouin zone. Black dotted line shows the Fermi level (*E*_f_). The metallic band structure, with large dispersion from *Γ* to *A*, is consistent with the large *t*_*a*_ (599 meV) along the *a* axis. Electrons are occupied below *E*_f_ in the density of states (DOS) (grey shadow). (b) Energy contour plots within the *ab* plane. The color scale indicates the energy from the Fermi level, with blue and red corresponding to *E* − *E*_f_ > 0 and < 0, respectively. Black solid and break lines display the Fermi surface indicating 1D (the *a*-axis) and 2D (the *ab* plane) metallic nature, respectively.

To gain further insight into the unique metallic properties derived from the stacked π-orbitals, we performed band structure analysis based on the crystal structure obtained from SXRD with density functional theory (DFT). The electronic band structure and Fermi surface confirm that the **TED** crystal is metallic with a large partially-filled bandwidth *W* > 1 eV near the Fermi level (Fig. 4). This provides additional evidence that the radical electrons extensively delocalize beyond each molecular unit. The large *W* derived from the energy dispersion is a consequence of the large transfer integrals along the *a*- and *b*-axes, namely a quasi two-dimensional (2D) electronic system (ESI, computational details†). Energy dispersion in a π-molecular system is generally expressed as a function of transfer integral in the tight-binding band model.^19^ We adopted DFT calculations here, but it is known to provide essentially the same results with tight-binding calculations employing the extended Hückel approximation.^23^ The anisotropy in the inter-**TED** transfer integrals is mirrored in the band structure, with a dispersive band along the *Γ* to *A* direction (*a*-axis), in contrast to a dispersionless one along the *Γ* to *Z* (*c*-axis). The Fermi surface is predominantly one-dimensional (1D) along *k*_*a*_ (*a*-axis) with a large open shape, reflecting *t*_*a*_ ≫ *t*_*b*_. A small closed Fermi surface appearing along *k*_*b*_ (*b*-axis) indicates weak 2D nature due to the finite value of *t*_*b*_ (Fig. 2b). A 1D metallic structure often causes a Peierls transition due to dimerization. Here, transverse intermolecular interactions suppress the distortion, which is a phenomenon that has been suggested in other molecular conductors,^19,11*b*^ and is consistent with the absence of a transition in our temperature-dependent measurements.

## Conclusions

We reported the structural and electronic properties of single-crystalline pure organic π-radical metal **TED** with high electrical conductivity over thousands S cm^−1^. A radical electron efficiently delocalizes in *k*-space through soft π-orbital overlap across multiple points, which affords large transfer integrals and a wide *W*, resulting in large *μ*. This demonstrates that strong intermolecular bonding, beyond van der Waals interactions, is not necessary to achieve metallic conduction in molecular crystals. Instead, two-dimensionally stacking of π-orbital is more critical for efficient electron transport in π-radicals. The findings greatly enhance the diversity of candidate molecules for practical use to form highly conductive crystals. Moreover, the *T*^3^-dependent *ρ* above *T* > 100 K implies a distinct mechanism for charge transport, with *T*-linear dependent simple metals possessing a spherical 3D Fermi surface. Our results show that highly conducting low-dimensional functional materials can be obtained from molecular radicals by π-orbital engineering.

## Experimental methods

### Sample preparation

**Fig. 5:**
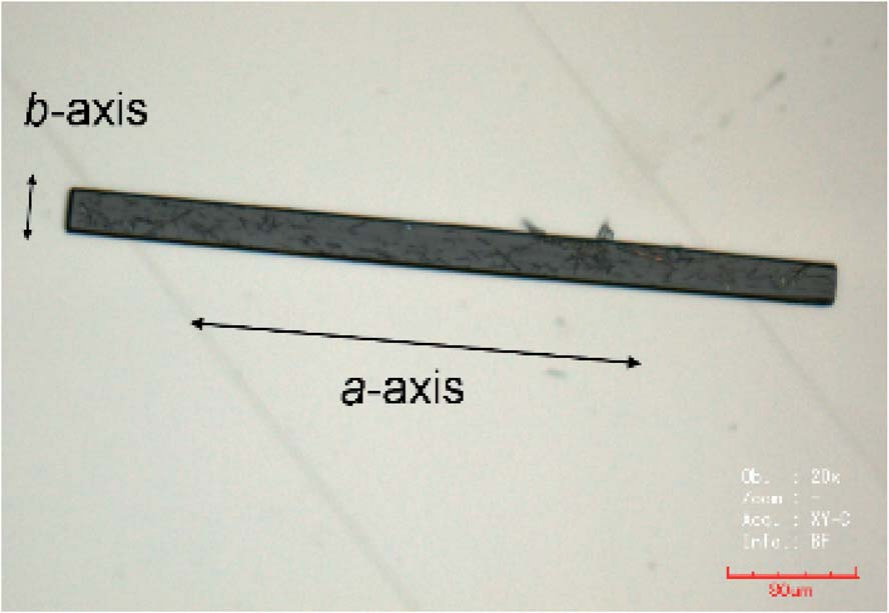
An optical microscope picture of the **TED** single crystal. The crystal long axis corresponds to the *a*-axis.

Dimethyl sulfoxide (DMSO) and hydroxylamine were purchased from Wako and Aldrich, respectively. **TED** was synthesized following the reported procedure.^12,13*b*^ Powdery crystal of **TED** (1 mg, 21 μmol) was ground in a mortar and solved in DMSO (160 μL). Hydroxylamine (50 wt% solution, 50 μL) was added into the solution in order to control acidity of the solution. (Here, hydroxylamine is not an oxidant. It enhances self-aggregation of **TED** molecules by adjusting p*K*_a_. Other basic compounds such as ammonia can play the same role.) The solution was kept in a closed vial container at RT for several months and then needle single crystals were obtained with a typical dimension of 400 μm × 20 μm × 10 μm (Fig. 5).

### X-ray crystallographic analysis

**Fig. 6:**
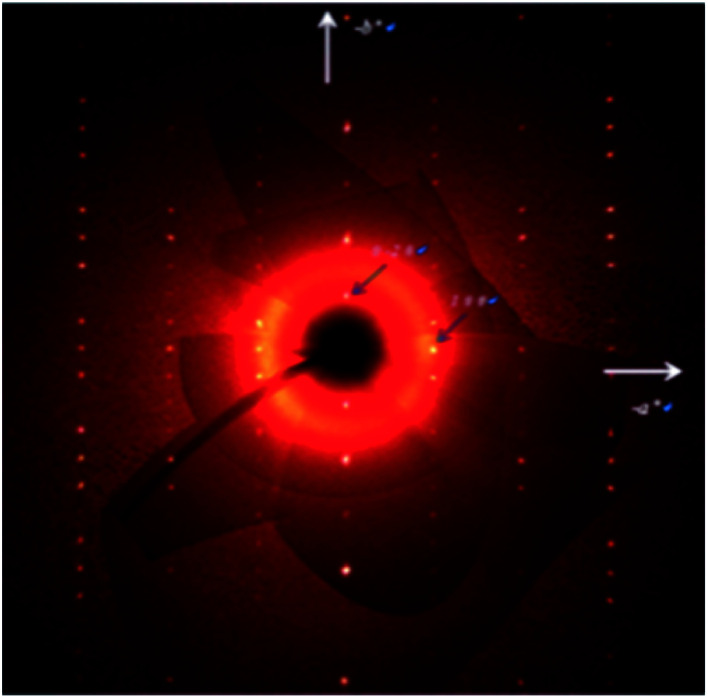
A typical X-ray diffraction image obtained for the **TED** single crystal.

Crystal structure analysis for a single crystal was carried out with using 1922 reflections with clear diffraction images (Fig. 6), which were collected at 113 K on a Rigaku Saturn CCD system. All atoms except hydrogen atoms were refined anisotropically.

### Resistivity measurements

Four probes were attached on the crystal with Au wire (0.01 mm ∅) using Ag paste (Dupont 4922N) as contact. Resistivity measurements were carried out on a sourcemeter (KEITHLEY 2450) and a nanovoltmeter (KEITHLEY 2182A) in a He cryostat from 305 to 2 K.

### Optical measurements

Raman spectra were measured using a longitudinal wave with a laser with 532 nm on a microscope Raman spectrometer (JASCO NRS-5500) at RT (ESI, Fig. S5†). Reflectance spectra was measured using P-wave on a microscope UV-Vis-NIR spectrometer (JASCO MSV-5200) and a microscope FT-IR spectrometer (FT/IR-JASCO 6700 type A) at RT. Reflectance of a standard Al mirror was used as a reference. We measured reflectance of Au on **TED** single crystal to assess an effect of roughness on absolute value of the reflectance (ESI, Fig. S6†). The sample was prepared with evaporation of Au (100 nm) on **TED** single crystal which was pre-coated with Ti (2 nm).

### Intermolecular transfer integrals calculations

The transfer integrals *t*, described as the electronic coupling matrix elements, were calculated using the fragment-orbital density functional theory (FODFT) method^24^ as implemented in the Car–Parrinello MD code (CPMD).^25^

### Electronic band structure calculations

The electronic band structure was calculated with DFT as implemented in VASP^26^ and plotted with the SUMO package.^27^ We employed the PBE functional revised for solids.^28^ Dispersion interactions were described *via* the DFT-D3 method.^29^ The electronic wave functions were expanded in a plane wave basis with kinetic energy cut-off of 600 eV and converged to 1 × 10^−5^ eV. The atomic forces were minimized to below 0.001 eV Å^−1^ and a *k*-point grid of 6 × 2 × 1 was used to sample the electronic Brillouin zone.

## Author contributions

Y. K. prepared the samples. Y. K. and K. H. measured transport properties. S. H., H. Y. and A. W. performed the calculations and theoretical analysis. Y. M. performed crystallographic measurements and analysis. K. I. and Y. K. performed spectroscopic measurements and analysis. Y. K. conceived the project and wrote the first paper draft. All authors contributed to the analysis and editing of the manuscript.

## Conflicts of interest

There are no conflicts to declare.

## Supplementary Material

SC-011-D0SC03521A-s001

SC-011-D0SC03521A-s002
