# Correction: Crystal structure and metallization mechanism of the π-radical metal TED

**DOI:** 10.1039/d0sc90231d

**Published:** 2020-10-22

**Authors:** Yuka Kobayashi, Kazuto Hirata, Samantha N. Hood, Hui Yang, Aron Walsh, Yoshitaka Matsushita, Kunie Ishioka

**Affiliations:** National Institute for Materials Science (NIMS) Sengen 1-2-1 Tsukuba Ibaraki Japan kobayashi.yuka@nims.go.jp; Department of Materials, Imperial College London Exhibition Road London SW7 2AZ UK a.walsh@imperial.ac.uk

## Abstract

Correction for ‘Crystal structure and metallization mechanism of the π-radical metal **TED**’ by Yuka Kobayashi *et al.*, *Chem. Sci.*, 2020, DOI: 10.1039/d0sc03521a.

The authors regret an error in Fig. 2e where the dotted line, acting as a guide to readers, was in the incorrect position. The corrected version is included here.

**Fig. 2:**
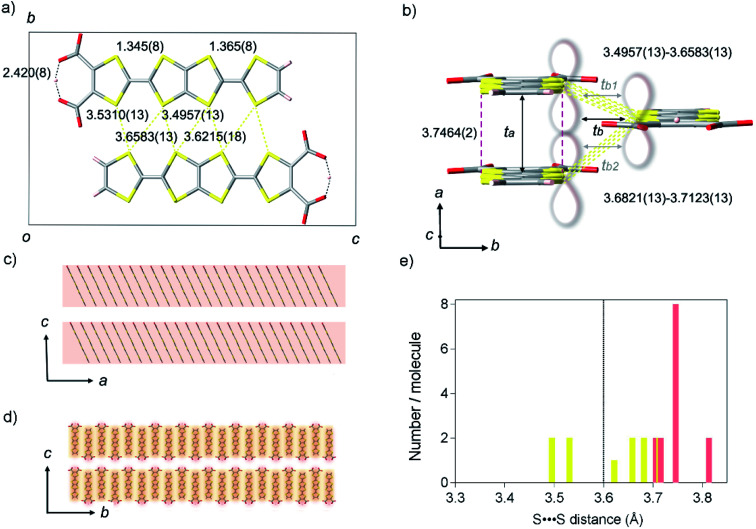
Crystal structure of **TED**. (a) A unit cell projected on the *a*-axis, represented with black square, consisting of two **TED** molecules with intermolecular distances of S⋯S contacts (yellow dotted line) and intramolecular hydrogen bond (black dotted line) in unit of Ångstrom. (b) A molecular stacking unit along the *a* and *b*-axes. Red break and yellow dotted lines show face-to-face and side-by-side intermolecular distances of S⋯S contacts, where π-orbital orbitals overlap longitudinally along the *a*-axis and transversely along the *b*-axis, respectively, as is guided with the lobe image. Black arrows display the transfer integrals *t*_*a*_ (599 meV), *t*_*b*_ (60 meV), which is averaged [*t*_*b*_ = (*t*_*b*1_ (69 meV) + *t*_*b*2_ (51 meV))/2]. (c and d) Super-structures of the **TED** molecular units crossing the *c*-axis; (c) stacking along the *a* direction; electric conduction occurs predominantly within the **TED** molecular layer, (d) stacking along the *b* direction. Red and yellow shadows show the anisotropic conduction pathways with *t*_*a*_ and *t*_*b*_, respectively, in contrast, inter-layer conductions along the *c*-axis are much lower with *t*_*c*_ ∼ 0. (e) Distribution of S⋯S distance in one molecule with an indication of van der Waals sum distance (black dotted line). Only one site was counted in the nearest neighbors with the same symmetry operation. Red and yellow bars show the contact along the *a*-axis and the *b*-axis, respectively.

The correction of Fig. 2e does not influence further discussion, or the conclusions of the article.

The Royal Society of Chemistry apologises for these errors and any consequent inconvenience to authors and readers.

