# Supplementary material for: Crystal structure and metallization mechanism of the π-radical metal TED
Source: Chem Sci. 2020 Sep 11;11(43):11699–704. doi: 10.1039/d0sc03521a (PMC8162741; doi:10.1039/d0sc03521a)
Supplement: SC-011-D0SC03521A-s001 [file SC-011-D0SC03521A-s001.pdf]

Electronic Supplementary Material (ESI) for Chemical Science.
This journal is © The Royal Society of Chemistry 2020

Supplementary Information

Crystal structure and metallization mechanism of the π-radical metal TED

Yuka Kobayashi[1*], Kazuto Hirata[1], Samantha N. Hood[2], Hui Yang[2], Aron Walsh[2*], Yoshitaka Matsushita[1], Kunie Ishioka[1]

1. National Institute for Materials Science (NIMS), Sengen 1-2-1, Tsukuba, Ibaraki, Japan
2. Department of Materials, Imperial College London, Exhibition Road, London SW7 2AZ, UK

Contents

1. Comparison between TED single crystal and self-standing film
2. Crystallographic details of TED single crystal
3. Raman spectrum
4. Reflectance spectra
5. Computational details

1. Comparison between TED single crystal and self-standing film

a) b)

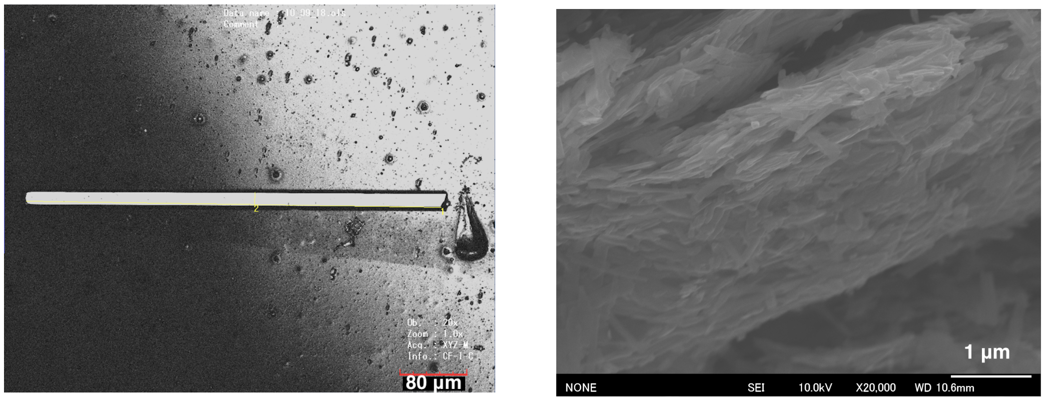

Fig. S1 Microscope pictures of TED crystals with a scale. a) Optical microscope image of a TED single crystal with μm size (this work), b) Scanning electron microscope (SEM) image for aggregation of TED polycrystals with nm size in self-standing film (ref. 12).

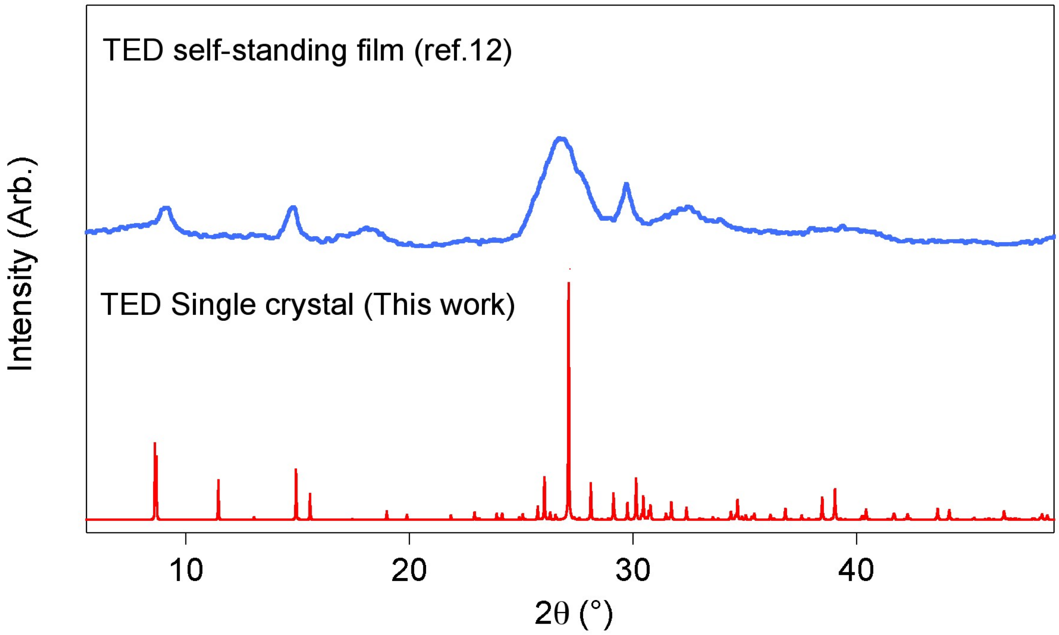

Fig. S2 Comparison of XRD patterns between TED single crystal (simulated by the X-ray single-crystal diffraction data at 113 K) and self-standing film (ref. 12).

2. Crystallographic details of TED single crystal

Table S1. Crystallographic data of TED single crystal

Formula	$C_{12}H_{2.5}O_4S_8$
MW	467.12
Crystal habit	Needle
Crystal system	Monoclinic
Space group	$P2_1/m$
a (Å)	3.7464(2)
b (Å)	11.8946(6)
c (Å)	20.2094(11)
β (°)	93.506 (2)
V (Å^3)	898.88 (8)
Z	2
GOF	1.154
Unique reflections	1922
Reflections[a]	1728
R_1 (%)	5.38
wR_2 (%)[b]	5.73
T (K)	113

[a] $I > 2\sigma(I)$. [b] All reflections were used.

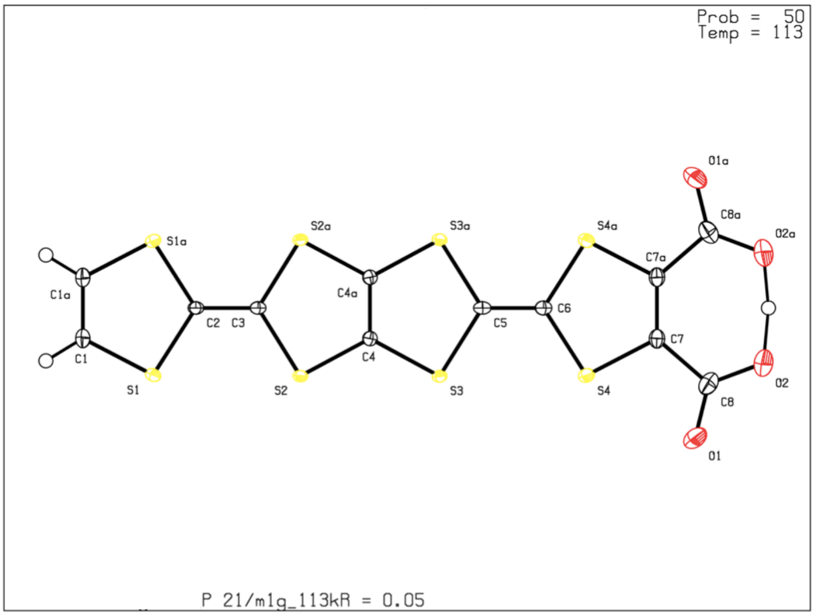

Fig. S3 Asymmetric unit of TED single crystal.

Table S2. Typical bond distances, angles and torsion angles.

Atom	Atom	Distance (Å)
C1	C1a	1.336(8)
C1	S1	1.741(4)
C2	C3	1.365(8)
C2	S1	1.747(3)
C3	S2	1.763(3)
C4	C4a	1.343(8)
C4	S3	1.739(4)
C4	S2	1.740(4)
C5	C6	1.345(8)
C5	S3	1.769(3)
C6	S4	1.755(3)
C7	C7a	1.354(8)
C7	C8	1.503(6)
C7	S4	1.745(4)
C8	O1	1.230(6)
C8	O2	1.292(6)
O2	O2	2.420(8)
C8a	O1a	1.230(6)
C8a	O2a	1.292(6)
O2	O2a	2.420(8)

Atom	Atom	Atom	Angle (°)	Atom	Atom	Atom	Atom	Torsion angle (°)
C1a	C1	S1	117.49(14)	C1a	C1	S1	C2	0.2(2)
C1	C1	C2	74.95(10)	S1a	C2	C3	S2	177.6(3)
S1	C1	C2	42.53(10)	S1	C2	C3	S2	-1.3(7)
S1a	C2	S1	114.8(3)	C2	C3	S2	C4	177.9(5)
S1	C2	C3	88.15(19)	S2	C4	S3	C5	-176.8(3)
C2	C3	S2	121.88(15)	C5	C6	S4	C7	179.5(5)
C3	S2	C4	67.07(17)	S4	C7	C8	O1	1.9(5)
S2	C4	S3	153.4(2)	S4	C7	C8	O2	-176.7(4)
S2	C3	S2	77.31(14)					
C4a	C4	S3	118.40(13)					
C4	S3	C5	93.6(2)					
S3	C5	C6	86.84(18)					
S3a	C5	S3	115.8(3)					
C6	S4	C7	68.20(18)					
S4	C6	S4	115.0(3)					
C7a	C7	C8	131.0(2)					
C7a	C7	S4	103.32(7)					
O1	C8	C7	117.1(4)					
O2	C8	C7	118.3(4)					
C8	O2	O2a	110.6(3)					

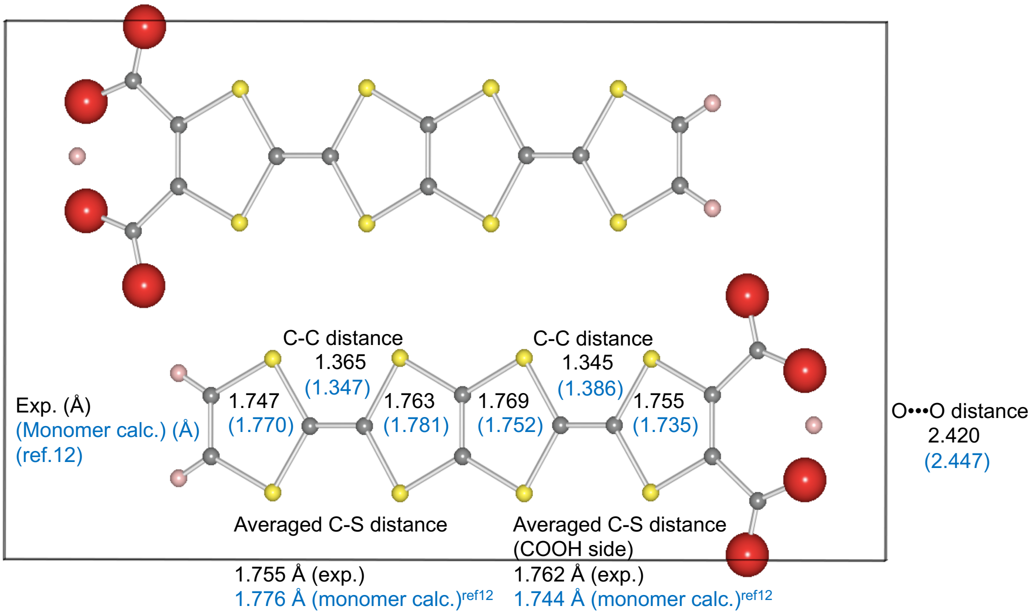

Fig. S4 Summary of key bond distances of TED single crystal (black) and previously calculated monomer (blue) (ref. 12). The difference in C-C and C-S distances between two TTF moieties is smaller than that in the monomer structure, which verifies delocalization of radical electron on the molecule.

3. Raman spectrum of single crystal TED

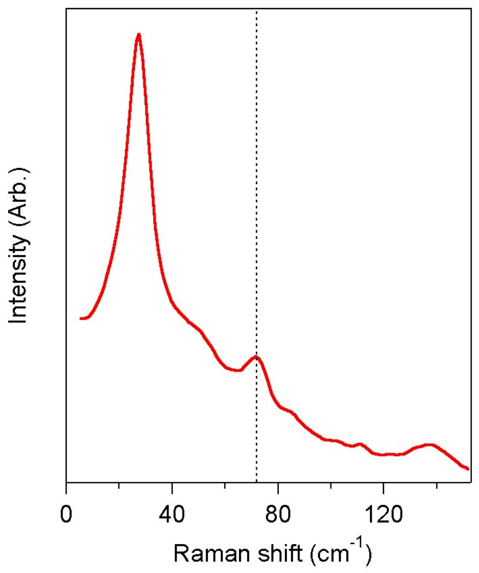

Fig. S5. Raman shift of the *ab* plane on TED single crystal in a low-frequency region below 150 cm^{-1} at RT. Black dotted line is at 72 cm^{-1}.

4. Reflectance spectra

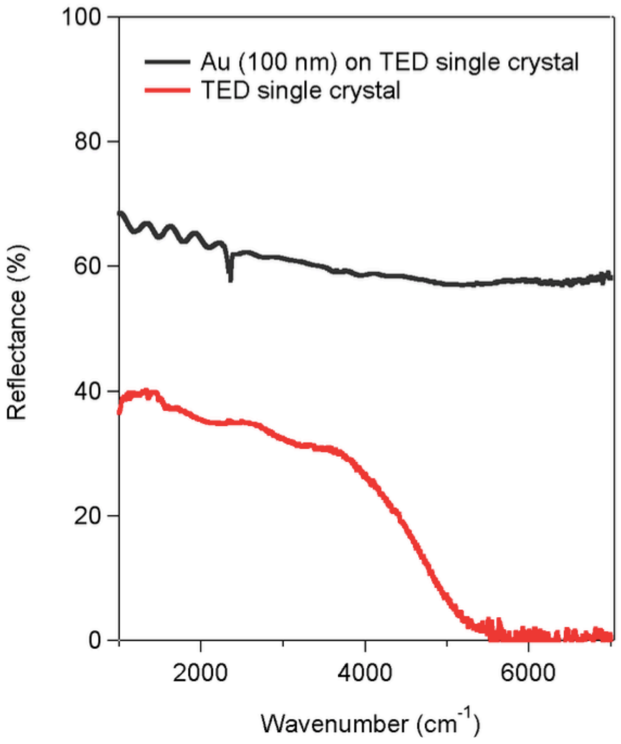

Fig. S6 Reflectance spectra of Au (100 nm) evaporated on TED single crystal and TED single crystal itself at RT. High reflectance in low wavenumber region below plasma edge is one of characteristics on traditional metals. However, many organic crystals have relatively rough surface, which normally affects the absolute value of the reflectance, different from mirror-like surface of the metals. Indeed, reflectance of Au evaporated on TED single crystal is decreased by ca 30 % from the reflectance of Au with mirror surface due to bearing the roughness of TED.

5. Computational details

5-1. Intermolecular transfer integrals

In the FODFT approach, first, two Kohn-Sham calculations for isolated TED molecules are carried out. φ_A and φ_B represent the singly occupied frontier wavefunctions of two nearest-neighbour TED molecules. The set of KS orbitals are then bi-orthogonalized and used for construction of the Kohn-Sham Hamiltonian (h^{KS}) of the total system. This method is extensively discussed in (ref.24), reviewed in (S1) and benchmarked in Ref. S2 and S3.

$$t = \langle \phi_A \; | h^{KS} | \phi_B \; \rangle$$

Calculations were carried out for the isolated system (no periodic boundary conditions) for the three distinct nearest-neighbour TED pairs found in the crystal environment. Here, the PBE exchange-correlation functional (S4) was used with a plane-wave cut-off of 90 Ry and GTH valence pseudopotentials as described in Ref. S5.

5-2. Electronic band structure

The role of spin-polarisation was tested but was not found to lower the total energy of the crystal compared to the spin-paired solution. The *k*-point mesh was set to 15x15 for mapping out the 2D dispersion in the *ab* plane and the *k*-points for the 1D dispersion diagram were 10 points in each segment along the symmetry path. The electronic structure and density of states were processed using the SUMO package (S2). The experimentally determined crystal structure (monoclinic, $P2_1$/m) was taken as the starting point for these simulations, which were then then relaxed to minimize their DFT (PBEsol+D3) forces. The hybrid HSE06 exchange-correlation functional was further tested and found not to change the metallic behavior of TED.

5-3. A simple model of quasi 2D band structure

In the tight-binding band approximation, quasi 2D system can be represented by a dominant electron transfer energy t_a and an additional interstack t_b. (ref.19)

$$\varepsilon(k) = 2t_a \bullet \cos(k_a \bullet a) + 2t_b \bullet \cos(k_b \bullet b)$$

$\varepsilon(k)$: energy dispersion, k_a (k_b): wave number of the *a*-axis (the *b*-axis), *a*, *b*: lattice spacing distance

References

[S1] Blumberger, *J. Chem. Rev*. **115**, 11191 (2015).

[S2] Kubas, A., Hoffmann, F., Heck, A., Oberhofer, H., Elstner, M., Blumberger, J. *J. Chem. Phys.***140**, 104105 (2014).

[S3] Kubas, A., Gajdos, F., Heck, A., Oberhofer, H., Elstner, M., Blumberger, J. *Phys. Chem. Chem. Phys.* **17**, 14342 (2015).

[S4] Perdew, J. P., Burke, K., Ernzerhof, M. *Phys. Rev. Lett.* **77**, 3865; *Phys. Rev. Lett.* **78**, 1396 (1997).

[S5] Goedecker, S., Teter, M., Hutter, J. *Phys. Rev. B* **54**, 1703 (1996).
